# Immediate Effects of Goldmann Applanation Tonometry on Central Corneal Thickness Measurements Using Contact and Non-contact Pachymetry Methods

**DOI:** 10.7759/cureus.76852

**Published:** 2025-01-03

**Authors:** Adiraj S Sibia, Raphael G Banoub, Isabel Eaddy, Anny Cheng, Aarup Kubal, Shailesh Gupta, K. V. Chalam

**Affiliations:** 1 Department of Ophthalmology, Florida Atlantic University-Broward Health North, Fort Lauderdale, USA; 2 Department of Ophthalmology, Florida Atlantic University-Broward Health North, Deerfield Beach, USA; 3 Department of Ophthalmology, Florida Atlantic University-Broward Health North, Philadelphia, USA; 4 Department of Ophthalmology, Your Eyes Specialists, Plantation, USA; 5 Department of Ophthalmology, Specialty Retina Center, Deerfield Beach, USA; 6 Department of Ophthalmology, Loma Linda University School of Medicine, Loma Linda, USA

**Keywords:** central corneal thickness, goldmann applanation tonometry, ophthalmology clinical practice, pachymetry, quality measurement

## Abstract

Purpose: The objective was to evaluate the immediate impact of Goldmann Applanation Tonometry (GAT), a gold-standard intraocular pressure measurement technique, on the reliability of central corneal thickness (CCT) measurements and to compare the outcomes between contact and non-contact pachymetry methods.

Methods: Fifty-one adult participants without ocular pathology were enrolled. Serial CCT measurements were conducted using three different pachymetry modalities across three days, both at baseline and post-GAT: Day 1 with an ultrasound pachymeter (USP), Day 2 using optical low-coherence reflectometry (OLCR), and Day 3 via anterior segment optical coherence tomography (AS-OCT). The study involved comparing mean CCT and CCT standard deviation at baseline and post-GAT using one-way analysis of variance (ANOVA) and paired t-tests.

Results: In the 610 CCT measurements performed, mean CCT did not show significant changes post-GAT overall or by modality (USP: +2.20 microns, p=0.63; OLCR: +1.0 microns, p=0.93; AS-OCT: +0.40 microns, p=0.95). However, contact USP consistently produced thicker corneal readings than non-contact OLCR and AS-OCT both at baseline and post-GAT (p < 0.0001). Notably, there was a significant increase in CCT standard deviation (+51.1%) and a decrease in adherence to technical repeatability specifications with contact USP following GAT (from 55% to 33%, p < 0.05).

Conclusions: The study suggests that contact intraocular pressure measurement methods like GAT may influence the reliability and repeatability of CCT measurements, as observed in our increased CCT measurement standard deviations. This could have important implications for accurate diagnosis and treatment planning for various ocular conditions. Given the study's limited sample size, further research may be warranted.

## Introduction

Pachymetry, the measurement of corneal thickness, holds a foundational role in ophthalmologic diagnostics. Central corneal thickness (CCT) measurements are essential for the assessment of various anterior segment ocular diseases, pre-operative planning for cataract and refractive surgeries, and the management of glaucoma. The process of pachymetry can be carried out using devices employing either contact or non-contact methods [1-4].

Clinical workflows and diagnostic testing algorithms within the field of ophthalmology may vary across practices and locations. However, a common practice involves conducting initial intake evaluations, including visual acuity, pupil examination, and intraocular pressure (IOP) measurement, prior to specific diagnostic examinations and tests to be performed for the day [5].

Goldmann applanation tonometry (GAT) stands as the gold standard method for measuring IOP. It involves applying a precise force to the anterior corneal surface, inducing the central corneal epithelium to align with the flat tip of the tonometer head [6,7]. By calibrating the user's input force with a prismatically refracted view of the tear film rings, GAT determines IOP. The extent of anterior corneal displacement posteriorly, and whether this is associated with corneal compression or transient rebound fluid accumulation within the corneal stroma, is not fully understood [8]. Nevertheless, when performing GAT, it is imperative to ensure that the tonometer makes contact with the central cornea rather than the peripheral cornea to obtain an accurate IOP reading.

IOP is not an independent indicator of ocular health. It is closely linked to, and may even be influenced by, various variables, including CCT [8,9]. Despite the potential for GAT to affect CCT measurements, there is a lack of empirical evidence concerning the immediate impact of conducting pachymetry shortly after GAT [10]. The objective of this study was to investigate the immediate effect of GAT on the mean and standard deviation (SD) of serial CCT measurements. We sought to determine whether these effects are more pronounced when utilizing contact pachymetry devices (Pachmate 2, DGH Technology, Exton, USA) in comparison to non-contact pachymetry devices (Lenstar LS 900, Haag-Streit Diagnostics, Koeniz, Switzerland, and OptoVue Inc., Fremont, CA, USA) [11,12]. Our hypothesis was that CCT measurement after GAT would exhibit a significant change in mean CCT and an increased SD of CCT measurements, thereby suggesting reduced reliability. Furthermore, we posited that these findings would be more significant in contact pachymetry modalities such as ultrasound pachymetry (USP) when compared to non-contact pachymetry modalities such as optical low-coherence reflectometry (OLCR) and anterior segment optical coherence tomography (AS-OCT).

## Materials and methods

Study participants

**Figure 1:**
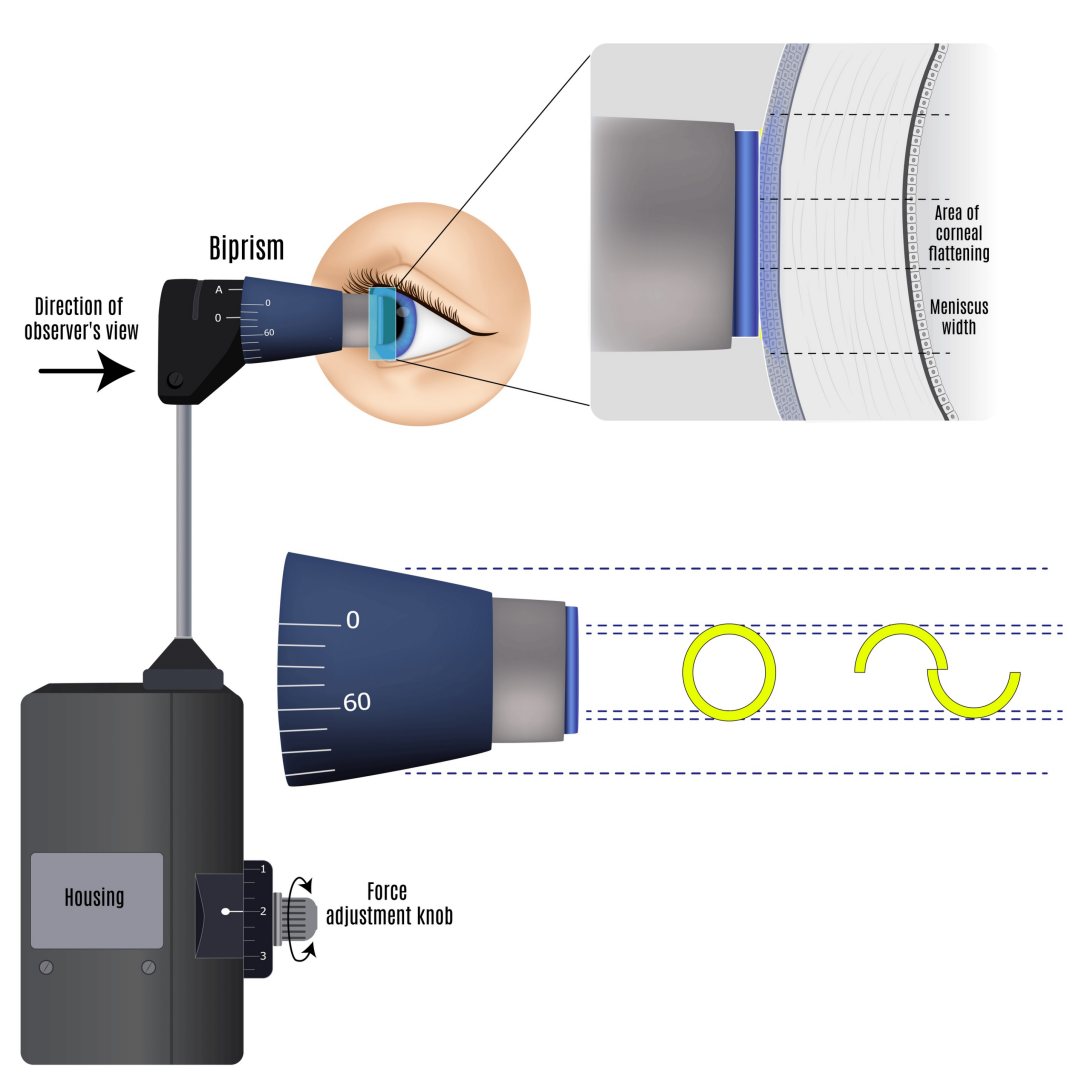
Goldmann applanation tonometry is based on the Imbert-Fick Principle, whereby the use of a 3.06 mm diameter tonometer head creates a condition in which the material resistance of the cornea equals the capillary action of the tear meniscus, thus enabling user measurement of intraocular pressure. Credit: The image was created by the authors.

Adults aged 18 years or older were prospectively recruited from a single healthcare organization's institutional directory. Informed consent was obtained for participation in this comparative study. Inclusion criteria included a corrected distance visual acuity (CDVA) of 20/20 or better and no ocular pathology, as confirmed by a comprehensive undilated ophthalmic examination. Contact lens wearers were required to abstain from lens use for at least 24 hours before examination. Exclusion criteria encompassed a history of ocular or oculoplastic surgery, any ocular pathology identified during examination, refractive errors exceeding a 6-diopter mean spherical equivalent, and IOP above 21 mmHg as measured by GAT as shown in Figure 1 [6,7].

Study design and data collection

**Figure 2:**
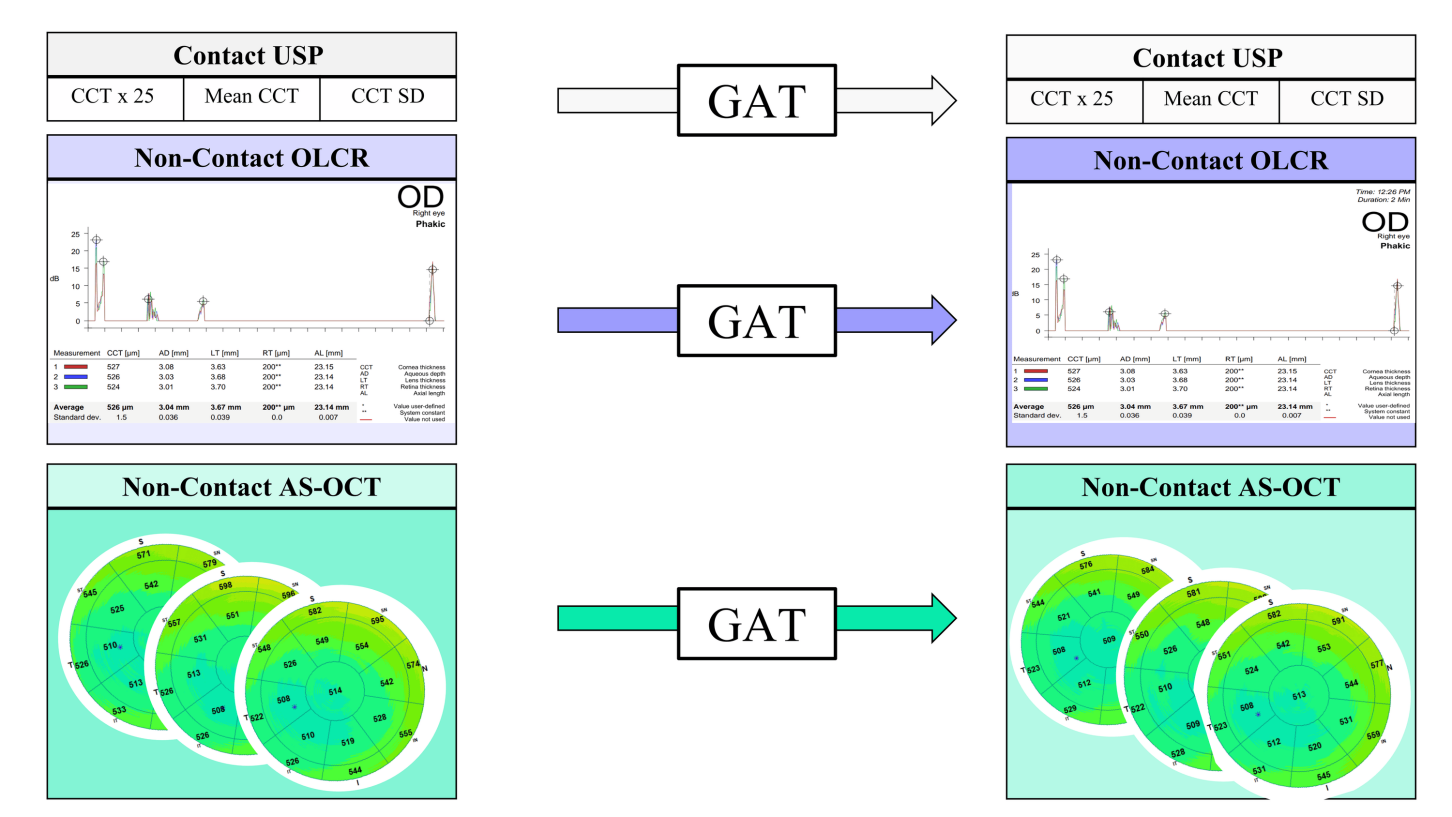
Study design: Participants received three serial CCT measurements on three consecutive days from 12:00 to 15:00, followed by GAT intraocular pressure measurement, followed by three serial CCT measurements using the same pachymetry modality. Key features of the reports from each pachymetry device can be reviewed: (top - contact ultrasound pachymeter (USP), middle - non-contact optical low-coherence reflectometry (OLCR), bottom - non-contact anterior segment optical coherence tomography (AS-OCT)) CCT: central corneal thickness; GAT: Goldmann applanation tonometry Credit: The image was created by the authors.

The study was structured as a prospective comparative investigation. Participants provided demographic details and underwent eligibility confirmation via a comprehensive 20-point ocular examination and CDVA measurement using a calibrated electronic Early Treatment of Diabetic Retinopathy Study (ETDRS) chart. Refractive error was assessed through analog lensometry. Eligible subjects received three sequential CCT measurements using one of three modalities, followed by GAT, and three additional CCT measurements. Each modality was assigned to a different day, with testing conducted between 12:00 and 15:00 over three consecutive days (Figure 2).

Devices

The DGH 55B Ultrasonic Pachymeter (USP) (Pachmate 2, DBH Technology) is a handheld device utilizing ultrasound waves to measure CCT. Measurements are triggered upon contact with the cornea, calculating thickness based on the time between acoustic echoes. The device provides individual measurements, mean CCT, and SD, focusing on central corneal measurements. Technical specifications include a range of 200-1100 µm, an accuracy of ±5µm, and a resolution of 1 µm [13].

OLCR, as utilized in the LenStar LS 900, employs low coherence interferometry for non-invasive CCT measurement. Light from a super luminescent diode creates interferometric signals from ocular reflections, which are resolved into axial scans [14,15]. This modality’s in-vivo repeatability for CCT includes a range of 300-800 µm, accuracy of 2.3 µm, and resolution of 1 µm.

The Avanti Widefield Anterior Segment Optical Coherence Tomography (OCT) (Optovue, Inc., VisionIX) utilizes widefield OCT with angiography, extending from the cornea to deeper ocular structures. The cornea and anterior chamber are imaged using a CAM lens adapter and 840 nm light sources, providing a color-coded pachymetry map. Technical specifications for healthy patients include a lateral resolution of 18 microns, a working distance of 20 mm, a scan depth of up to 3 mm, and a scan length of 2-18 mm. Repeatability is noted at 2.1 microns with a resolution of 1 micrometer [16-21].

Statistical analysis

Data analysis was conducted using GraphPad Prism 9 (GraphPad Software, San Diego, USA). Participants underwent three CCT measurements before and after GAT across three consecutive days using each modality. Mean CCT and SD were calculated for each participant before and after GAT. Univariate analysis followed by one-way analysis of variance (ANOVA) was employed to compare CCT readings across modalities, both at baseline and after GAT. A two-tailed unpaired t-test assessed the reliability differences in CCT measurements between contact and non-contact modalities.

## Results

Study demographics

A total of 56 healthy adult volunteer participants were recruited for the study, however, five participants were excluded due to a history of refractive surgery (three participants), recent contact lens use (one participant), and tilted optic disc (one participant). All other participants (n=51) met the inclusion criteria. The mean age was 26.63 years (SD: 6.84). The gender distribution was 49% female and 51% male. Racial identification of participants included eight identifying as Black, 13 identifying as Hispanic or Latino/a, 16 identifying as White/Caucasian, and 14 identifying with other racial backgrounds. Smoking status revealed 42 never smokers, six former smokers, and three current smokers. Alcoholic beverage consumption showed that 13 participants (25%) reported never consuming alcohol, while 38 (75%) reported occasional consumption (1-3 beverages/week). Contact lens usage was noted in six participants (12%), albeit outside the 24-hour window prior to CCT measurement. The mean spherical equivalent refraction for spectacles wearers was -0.90 (SD 1.40), and the average IOP was 12.98 mm Hg. The study's demographic information is shown in Table 1.

**Table 1:** Study demographics D: diopter; Y: years old

Participant Demographics and Characteristics	
Total Number of Participants	51.00
Age (Y)	26.3 ± 7.3
Gender	
Male	25 (49.0%)
Female	26 (51.0%)
Race	
Black	8 (15.7%)
White	16 (31.3%)
Hispanic/Latino	13 (25.5%)
Other	14 (27.5%)
Mean Spherical Refractive Equivalent (D)	-0.9 ± 1.4
Tobacco Use	
Current smoker	42 (82.3%)
Former smoker	6 (11.8%)
Never smoker	3 (5.9%)
Alcohol Use	
Occasional	38 (74.5%)
Never	13 (25.5%)
Contact Lens Wear	6 (11.7%)

Pachymetry: mean CCT

Baseline and After-GAT CCT Measurements

CCT was measured in 610 participants using USP, OLCR, and AS-OCT. Initial assessments revealed a mean baseline CCT of 557.7 microns (USP: 569.1, OLCR: 552.7, AS-OCT: 545.3 microns). After-GAT measurements showed a slight increase to 558.6 microns (USP: +2.20 microns, p=0.63; OLCR: +1.0 microns, p=0.93; AS-OCT: +0.40 microns, p=0.95), however this was not statistically significant. ANOVA tests indicated significant differences in CCT measurements between the modalities such that USP consistently registered thicker measurements both at baseline (p<0.01) and after-GAT (p<0.001). Mean CCT results are shown in Table 2.

**Table 2:** Pachymetry mean central corneal thickness CCT: Central corneal thickness; GAT: Goldman applanation tonometry; USP: Ultrasound pachymeter; OLCR: Optical low-coherence reflectometry; AS-OCT: Anterior segment-optical coherence tomography; ANOVA: Analysis of variance

Measurement Group			
N=610	Mean CCT (µm)		
	At Baseline	After GAT	∆CCT
Overall	557.7	558.6	+0.9 (ns)
USP	569.4	571.6	+2.2 (ns)
OLCR	552.7	553.7	+1.0 (ns)
AS-OCT	545.3	545.7	+0.40 (ns)
ANOVA	p <0.01	p <0.001	
Contact (USP)	569.1	571.6	
Noncontact (OLCR, AS-OCT)	549.0	549.7	+0.7 (ns)
T-Test	p <0.05	p <0.001	

Comparative Analysis of Pachymetry Modalities

This study also compared contact (USP) and non-contact (OLCR/AS-OCT) pachymetry methods and found that contact pachymetry consistently reported thicker CCT measurements compared to non-contact methods, both at baseline (p < 0.01) and after-GAT (p < 0.001).

Immediate Impact of GAT on CCT

A two-tailed, paired t-test was used to assess the immediate effect of GAT on CCT. The analysis revealed no significant change in the overall mean CCT after-GAT (baseline: 557.7, after-GAT: 558.6, +0.91 microns, p=0.81). This non-significant change was consistent across all pachymetry modalities.

Pachymetry: CCT SD

Baseline and After-GAT CCT SD

The study also analyzed CCT's SD at baseline and following GAT, utilizing data directly from each pachymetry device report. Baseline CCT SD averaged 3.05 microns and increased to 3.87 microns after-GAT. CCT SD results are shown in Table 3.

**Table 3:** Pachymetry central corneal thickness standard deviation CCT: Central corneal thickness; GAT: Goldman applanation tonometry; USP: Ultrasound pachymeter; OLCR: Optical low-coherence reflectometry; AS-OCT: Anterior segment-optical coherence tomography; ANOVA: Analysis of variance

Measurement Group	CCT Standard Deviation (µm)		
	At Baseline	After GAT	∆SD
Overall	3.05	3.87	+0.82 (ns)
USP	5.32	7.21	+1.89 (p <0.05)
OLCR	1.86	2.59	+0.73 (ns)
AS-OCT	1.22	0.89	-0.33 (p <0.05)
ANOVA	p <0.0001	p <0.0001	
Contact (USP)	5.32	7.21	
Noncontact (OLCR, AS-OCT)	1.53	1.73	+0.2 (ns)
T-Test	p <0.0001	p <0.0001	

Comparative Analysis of Pachymetry Modalities

ANOVA by modality revealed that USP consistently registered a significantly higher CCT SD than OLCR and AS-OCT both at baseline and post-GAT (p < 0.0001 for all comparisons). Specifically, contact pachymetry (USP) demonstrated elevated CCT SDs before GAT (5.32 microns) and after GAT (7.21 microns) compared to the combined non-contact modalities (OLCR/AS-OCT) (baseline: 1.53 microns, after-GAT: 1.73 microns, p < 0.0001).

Immediate Impact of GAT on CCT SD

While the aggregate data suggests no significant change in CCT SD after-GAT across all modalities, there were noteworthy modality-specific changes. USP exhibited an increase (from 5.32 to 7.21, +1.89, p < 0.05), AS-OCT exhibited a decrease (from 1.22 to 0.89, -0.33, p < 0.05), and OLCR exhibited no significant change in CCT SD after GAT (p < 0.05).

Percent Change in CCT SD and Repeatability

This study observed a +43.3% increase in CCT SD after GAT compared to baseline. USP showed the largest increase in CCT SD at 51.1%. Furthermore, this study examined interval changes in adherence to manufacturer-specified technical repeatability standards after-GAT. A significant decline in adherence to technical specifications was noted with USP after-GAT (from 52% to 33%, p < 0.05). At baseline and after-GAT, the adherence to technical specifications for repeatability was lowest at USP, followed by OLCR, and greatest with AS-OCT at baseline (p < 0.0005) and after-GAT (p < 0.0001). Contact pachymetry demonstrated significantly lower adherence to technical specification for repeatability compared to non-contact methods, both at baseline and after-GAT (p < 0.0001 for all). CCT reliability results are down in Table 4.

**Table 4:** Pachymetry central corneal thickness technical specifications for repeatability CCT: Central corneal thickness; GAT: Goldman applanation tonometry; USP: Ultrasound pachymeter; OLCR: Optical low-coherence reflectometry; AS-OCT: Anterior segment-optical coherence tomography; ANOVA: Analysis of variance

Measurement Group	Technical Specification: Repeatability Adherence (%)		
	At Baseline	After GAT	∆Repeatability (%)
Overall	74%	64%	-12% (ns)
USP (<5 µm)	55%	33%	-19% (p <0.05)
OLCR (<2.3 µm)	74%	63%	-11% (ns)
AS-OCT (<2.1 µm)	97%	97%	0 (ns)
ANOVA	p <0.0005	p <0.0001	
Contact (USP)	55%	33%	
Noncontact (OLCR, AS-OCT)	86%	80%	-6% (ns)
T-test	p <0.0001	p <0.0001	

## Discussion

CCT is an essential indicator of corneal health and serves as a key indicator for a range of ocular diseases, including cataracts, glaucoma, and anterior segment diseases, particularly those necessitating surgical correction of refractive errors such as myopia, hyperopia, and astigmatism [22-27]. In refractive surgery, precise CCT management is essential for optimal outcomes [23]. Prior to cataract surgery, CCT evaluation is imperative to ensure the safe administration of phacoemulsification energy and to assess the need for concurrent corneal surgical interventions. CCT is also further emerging as a prognostic factor in corneal dystrophies such as Fuchs endothelial dystrophy and is ever more crucial in measurement during pre-surgical evaluations [25-27].

The intricate relationship between CCT, ocular hypertension, and glaucoma plays a crucial role in the clinical decision-making process for ophthalmologists [24-26]. Accurate IOP measurement via GAT, and consequent glaucoma management, relies heavily on a CCT within a normal distribution [25,26]. Historical models have suggested the use of correction formulas for IOP readings to account for CCT variability among patients, although this is still debated in the field [25,26]. Further complicating this dynamic, recent studies highlight a significant correlation between reduced CCT and the progression of advanced glaucoma underscoring a significant association between diminished CCT and advanced glaucoma, underscoring the critical and potentially reciprocal relationship between CCT and IOP. Finally, chronic severely elevated IOP can precipitate corneal conditions such as decompensation, edema, or ectasia, leading to substantial and varied changes in CCT among different patients [27].

In standard ophthalmology practice, initial evaluations include vision, pupillary examination, and IOP measurement. The gold standard for measuring IOP is GAT, which works based on the Imbert-Fick principle of mechanical compression of a thin-walled sphere. When operated, the tonometer (diameter: 3.06 mm) refracts the user’s view of the tear meniscus, held to the tonometer by surface tension, into two horizontally juxtaposed semicircular rings (mires) [6,7]. Calibration of diminutive tonometer head force brings the flat tonometer head in alignment with the anterior cornea. Ultimately, it is the mathematical assumption that the capillary action of the tear meniscus at a diameter of 3.06 mm equals the material resistance of the anterior cornea to posterior displacement that IOP is determined. However, it remains unclear where posterior displacement of the central cornea is also associated with varying degrees of compression of the central corneal tissue.

Our study focused on the immediate impact of GAT on CCT measurements in a population without ocular disease, using both contact and non-contact pachymetry modalities. While previous research primarily compared equipment reliability, our study sought to explore the mutual confounding effects of IOP measurement via GAT immediately prior to CCT measurements. Our findings indicate that while mean CCT did not significantly change post-GAT overall or per modality (USP, OLCR, AS-OCT), CCT readings with contact USP were consistently thicker than either non-contact (OLCR or AS-OCT) modality at baseline and post-GAT (p <0.001) and observed increases in CCT SD and decreases in reliability after-GAT were more pronounced with contact USP than with either non-contact (OLCR or AS-OCT) pachymetry modality individually or collectively. When reliability was reviewed in the context of technical specifications of repeatability, we again found that the specification adherence was 1.56x lower with contact USP pachymetry at baseline (55% vs 86%, p <0.0001) and 2.42x lower after-GAT (33% vs 80%, p <0.0001), compared non-contact modalities. Furthermore, we found that there was a significant interval reduction in technical specification adherence for repeatability after-GAT with contact USP (19% interval reduction, p <0.05 but no significant findings were observed for the non-contact (OLCR/AS-OCT) modalities.

**Figure 3:**
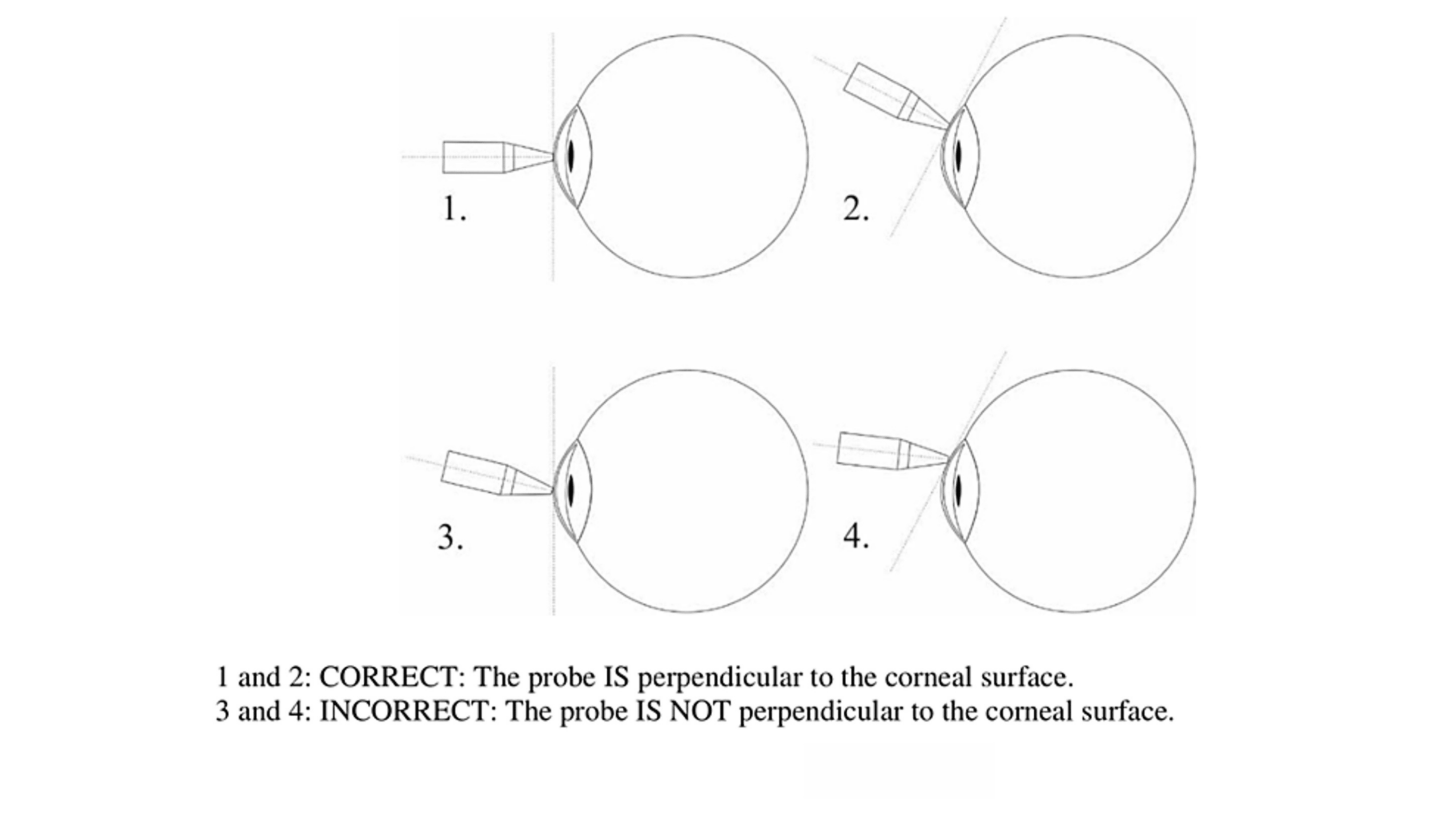
DGH 55 MHz Pachmate2 User Manual: Proper applanation during contact USP occurs when the flat tip of the probe comes into full contact with the cornea perpendicular to the cornea surface. Users must ensure the probe tip is not moved or realigned while in contact with the cornea and that pressure is not applied so to not damage the cornea. USP: Ultrasound pachymeter Credit: Reference [13]

The observed discrepancies in CCT readings post-GAT, particularly with contact pachymetry, suggest potential topographical changes in the central cornea during GAT, possibly leading to misalignments in pachymeter probe contact as shown in Figure 3. Additionally, differences in measurements could be influenced by alterations in the tear meniscus after the application of anesthetic drops for GAT, which may affect probe positioning and accuracy [13].

These findings highlight the need for cautious interpretation of CCT measurements post-GAT, especially when using contact pachymetry methods. Given the prevalent use of GAT and CCT measurements in ophthalmology clinics worldwide, these results underscore the importance of considering potential measurement variances in clinical practice. The limitations of this study include a small sample size and the restriction to only three serial CCT measurements per participant for each modality. While the observed CCT variations were generally small, their clinical relevance cannot be understated given the routine nature of GAT and CCT assessments in ophthalmology. Future research with larger sample sizes and more extensive serial measurements is warranted to further elucidate the interplay between GAT and CCT measurements.

## Conclusions

Our study demonstrates that GAT can impact the reliability of CCT measurements, particularly when using contact USP. While the mean change in CCT post-GAT was minimal (approximately ±0-3 microns), contact USP consistently showed thicker measurements and greater variability compared to non-contact methods. Although these differences may not be clinically significant on their own, they highlight the potential for cumulative effects on diagnostic accuracy and patient management. The contact nature of USP, along with possible probe misalignment or changes in the tear film after anesthetic drops, may contribute to subtle measurement inconsistencies. Clinicians may benefit from considering these secondary effects of GAT, particularly when using contact modalities. Further research with larger sample sizes is warranted to confirm these findings and inform refinements to clinical workflows.
